# Evaluating the Use of a Robot in a Hematological Intensive Care Unit: A Pilot Study

**DOI:** 10.3390/s23208365

**Published:** 2023-10-10

**Authors:** Michela Falcone, Grazia D’Onofrio, Giuseppina Iannacone, Matteo Steduto, Angelo Michele Carella, Francesco Giuliani, Francesco Ricciardi

**Affiliations:** 1 Research and Innovation Unit, Fondazione IRCCS Casa Sollievo della Sofferenza, 71013 San Giovanni Rotondo, Italy; m.falcone@operapadrepio.it (M.F.); g.iannacone@operapadrepio.it (G.I.); f.giuliani@operapadrepio.it (F.G.); f.ricciardi@operapadrepio.it (F.R.); 2 Health Department, Clinical Psychology Service, Fondazione IRCCS Casa Sollievo della Sofferenza, 71013 San Giovanni Rotondo, Italy; 3 Haematological Intensive Care Unit, Fondazione IRCCS Casa Sollievo della Sofferenza, 71013 San Giovanni Rotondo, Italy; m.steduto@operapadrepio.it (M.S.); am.carella@operapadrepio.it (A.M.C.)

**Keywords:** robotics, assistive robotics, intensive hematological unit, logistics, surveillance, robot entertainment, remote visits, psychological evaluation

## Abstract

The aim of the SYRIACA project was to test the capability of a social robot to perform specific tasks in healthcare settings, reducing infection risks for patients and caregivers. The robot was piloted in an Intensive Hematological Unit, where the patients’ and healthcare operators’ acceptability of the robot was evaluated. The robot’s functions, including logistics, surveillance, entertainment, and remote visits, were well accepted. Patients expressed interest in having multiple interactions with the robot, which testifies to its engaging potential and that it provides useful services. During remote visits, the robot reduced perceived stress among patients, alleviating feelings of isolation. The successful implementation of the robot suggests its potential to enhance safety and well-being in healthcare settings.

## 1. Introduction

SARS-CoV-2 (Severe Acute Respiratory Syndrome Coronavirus 2) is a disease that has recently been caused by zoonic coronaviruses [1]. Like SARS-CoV (severe acute respiratory syndrome coronavirus) and MERS-CoV (Middle East Respiratory Syndrome Coronavirus), which also developed in the last two decades, SARS-CoV-2 originated from bats [2] and affects humans. The COVID-19 pandemic started at the end of November 2019, causing a great number of human losses at the global level.

Thanks to the development of vaccines, the number of deaths has drastically decreased [3], even if the virus remains a public health threat and new mutations of the virus are increasingly being generated due to its diffusion among humans. This spread can occur mainly in three ways that are not mutually exclusive: (i) airborne transmission via respiratory droplets and aerosols; (ii) direct contact where the virus is deposited on people; and (iii) indirect contact when the virus is transmitted via objects [4].

While 81% of COVID-19 patients have mild to moderate symptoms, approximately 14% have symptoms that may require hospitalization in intensive care units, and the remaining 5% develop more critical symptoms, including septic shock and multi-organ dysfunction or failure [1]. Host factors that can influence the clinical outcome are, for example, age, gender, pregnancy, comorbidity, and frailty [5]. Patients affected by circulating tumors and hospitalized in Hematological Intensive Care Units are more prone to fatal complications [6].

During the pandemic crisis, physicians, nurses, and, more generally, caregivers found themselves in dangerous situations while carrying out their work [7]. The activities performed in contact with patients affected by COVID have jeopardized the physical and mental health of medical staff, who have been at risk of infection and/or of becoming hosts for the virus [8].

The goal of the SYRIACA project was the experimentation of a robot capable of working side by side with healthcare operators and taking care of more repetitive tasks, thus reducing the exposure of both operators and patients to infection risks. The robot was tested in different use cases and at four pilot sites: Belharra Clinic in Bayonne (France), Humana Clinic in Palma de Mallorca (Spain), Fondazione IRCCS Casa Sollievo della Sofferenza in San Giovanni Rotondo (Italy), and Aktios in Agia Paraskevi (Greece) [9].

In this paper, we present and analyze the results of the experimentation on the acceptability of the robot at the pilot site, Fondazione IRCCS Casa Sollievo della Sofferenza.

## 2. Materials and Methods

This study was conducted in compliance with the Declaration of Helsinki, the Guidelines for Good Clinical Practice, and the Guidelines for Strengthening the Reporting of Observational Studies in Epidemiology and approved by the local Ethics Committee for human trials. It was a pilot study in which the assignment of a participant to a specific intervention or control arm, the following assessment of effects, and biomedical or behavioral health-related outcomes were not considered.

The robot used in the pilot activities is based on a robotic platform manufactured by Kompai Robotics, Anglet, France. Kompai, which was awarded by the DIH-HERO European initiative, is the project coordinator. The robot is characterized by the following elements: (a) four wheels, of which two simple and non-steering active side wheels, a castor passive front wheel and a castor passive rear wheel; (b) a fisheye camera for the recognition of fallen people at a distance of less than 3 m from the robot; (c) a headband camera which has the dual purpose of generating the video streamed on the tablet behind the robot’s head and of recognizing people sitting or standing at a distance shorter than 6 m from the robot; (d) two shelves for carrying small items; (e) LED lights whose color determines the distance from obstacles; (f) the tablet behind the robot’s head for functions management; (g) two LIDARs at different heights to detect obstacles.

**Figure 1:**
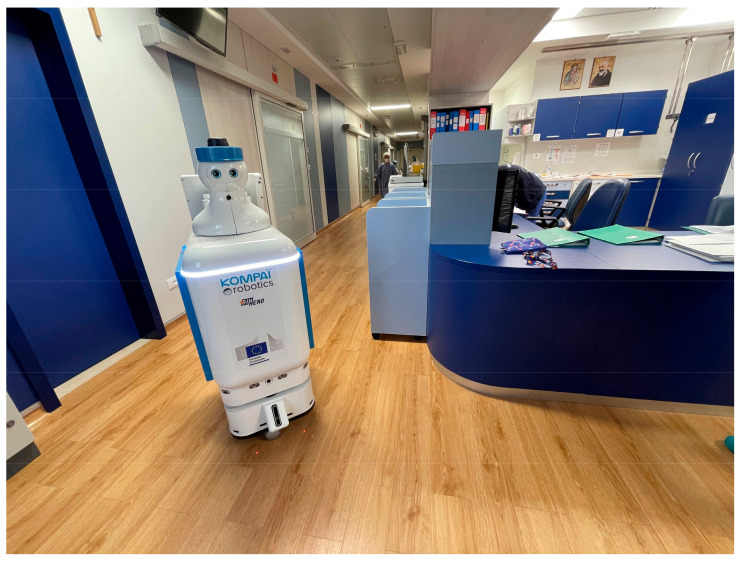
The robot within the ward.

The robot was used in the Hematological Intensive Care Unit, Figure 1, where hospitalized patients are characterized by a high level of frailty and must be isolated for the entire duration of their stay, while also keeping contact with the medical staff to a minimum.

The project can be divided into two phases according to the functions performed by the robot. The first phase concerned logistics, surveillance, entertainment, and the detection of vital parameters of patients with two smartwatches, such as temperature, pressure, oxygen saturation, and heart rate. It took place from January 2023 to March 2023. The second phase, which took place in April 2023, allowed patients to make video calls with family members, psychologists, and nutritionists. Both phases were approved separately by the local Ethics Committee (Prot. Nos. 719/D6/2023 and 1153/01-D4).

Three applications are available on the tablet: the Toolbox app, entertainment tours, and surveillance tours. A map of the ward was created and uploaded through the Toolbox app. The robot can be located on the map by scanning a QR code or by manually selecting, on the map, the point where the robot is. POIs (Points of Interest) were created on the map, i.e., points through which the robot must pass and stop for a certain period of time with a certain orientation. To verify that the robot is able to arrive at a new point of interest, the “move to” command is used. In total, twelve POIs have been created within the ward, six of which are at the entrance to the patient rooms. We used the “move to” command for logistical purposes, i.e., to move small objects both to a POI or to another manually selected point on the map. An ordered list of POIs for which the robot must successfully arrive and stop for a specific period is called a “tour”. Tours are used for autonomous driving during entertainment and surveillance functions.

With the entertainment function, the robot, in autonomous driving mode, can broadcast music or announcements. Announcements can be added from the settings panel; the frequency with which they are broadcast can be defined (we decided to broadcast announcements every 2 min). During the entertainment tour, the robot can also be used to distinguish people who do not wear masks and to recognize fallen people. In the first case, the robot sends a message to the user, while in the second case, it produces a sound alert. 

During the surveillance function, the robot must follow the selected tour for a certain period of time. The robot can be asked to perform one or more of the following tasks: (a) to distinguish people who wear a mask from those who do not; (b) to recognize fallen people; (c) to detect people; (d) to stop when it detects a person. These last two functions were almost never used.

For the measurement and monitoring of vital parameters, two smartwatch prototypes from Healysa (France) were used. The measurements were requested two or three times a day through the Healysa web page, in the “My Health” section. The resulting parameters were compared with those obtained using medical devices currently used in clinical practice.

In the first part of the study, we followed the following procedure to recruit patients:

(a) explained the project to the patients and collected informed consent from them;
(b) delivered the smartwatch to the patients;
(c) administered psychological tests aimed at assessing the psycho-physical health of the patient: the Neuropsychiatric Inventory (NPI), which is based on a structured interview with a caregiver and/or patient’s relative. The following 12 neuropsychiatric domains were evaluated: delusions, hallucinations, agitation/aggression, depression, anxiety, euphoria, apathy, disinhibition, irritability/lability, aberrant motor activity, sleep disturbances, and eating disorders. For each domain, the robot asks a screening question to determine whether the behavioral change is present or absent. If the answer is positive, then the domain is explored more in depth with sub-questions. If the sub-questions confirm the screening question, frequency is rated from 1 to 4 and severity is scored from 1 to 3. The product (severity x frequency) is calculated for each behavioral change occurring during the previous month or since the last evaluation. Patients with NPS were identified on the basis of the following parameters: presence of delusions or hallucinations on the NPI (i.e., a score of ≥ 1 on either subscale, and/or dysphoria score > 6, anxiety score > 6, disinhibition score > 4, irritability/lability score > 2, and/or score on the apathy, agitation/aggression, euphoria, aberrant motor behavior, sleep disturbance, and eating disorder subscale > 1), the Hamilton Rating Scale for Depression (HDRS), and the European Quality of Life 5 Dimensions 3 Level Version (EQ-5D-3L);
(d) interaction with the robot;
(e) administration of tests to evaluate the experience with the robot: Almere Model Questionnaire (AMQ), System Usability Scale (SUS), GODSPEED, and User Experience Questionnaire (UEQ).

Eight patients, three women (37.5%) and five men (62.5%), were involved in the first part of the study. They were aged between 19 and 66 years, average age 44.1, std. dev 18.12. Inclusion criteria were based on age (between 18 and 70 years), length of hospitalization (not less than 14 days), and capability to understand informed consent. 

During the second part of the project, the recruitment procedure was as follows:

(a) explanation of the project goals and a request to sign informed consent;
(b) selection of the most appropriate visit with the psychologist, dietician, or family member;
(c) execution of the remote visit;
(d) administration of the Perceived Stress Scale (PSS), SUS, and UEQ questionnaires to the patient;
(e) administration of questionnaires to healthcare operators and family members: Unified Theory of Acceptance and Use of Technology (UTAUT), PSS, and UEQ.

The patients enrolled in the second phase were 4:3 men (75%) and 1 woman (25%) aged between 50 and 66 years, average 60.2, std. dev. 7.13. They did not participate in the first part of the project. Table 1 reports the patients’ age distribution in both phases.

## 3. Results

In the first part of the project, each interaction between the patients and the robot lasted about 45 min. During the interactions, all patients expressed curiosity about the various functions of the robot, especially the entertainment function. The most frequently expressed request was to add some custom announcements and hear the robot speak. The behavior of the robot in the presence of obstacles and the possibility of guiding it via the joypad inside the room also aroused curiosity. Most patients wanted to interact more than once with the robot; however, two out of eight patients showed no interest in having a second interaction.

The patients used the smartwatches for vital parameters monitoring over the following periods of time (Table 2):

Therefore, three patients (23-1, 23-5, and 23-6) used the smartwatch for three weeks, two patients (23-3, and 23-4) for two weeks, and the remaining three patients (23-2, 23-7, and 23-8) for one week.

During the second part of the study, a total of six remote visits were made with the robot, distributed as shown in Table 3. Each remote visit lasted approximately 15 min.

Patient 23-9 used the robot for two remote visits: one with the dietician and one with the psychologist. Patient 23-10 used the robot only for the visit with the dietician. Patient 23-11 had two visits: one with the family and one with the psychologist. Patient 23-12 only had a visit with the family.

At the end of each remote visit, 3 out of 4 patients were curious about the other functions of the robot and about the first part of the project.

In the first part of the study, as shown in Table 4, the patients did not report neuropsychiatric symptoms since they presented a low NPI average score and/or affective disorder due to the low HDRS average score. The low aggregated value of EQ-5D-3L also shows they had a good quality of life and a good level of perception of their health based on the score of EQ VAS.

As shown in Table 5, after the interaction with the robot, the participants confirmed a high level of acceptability in the AMQ domains: low level of anxiety (ANX = 7.59 average value), good attitude (ATT = 12.25 average value), few facilitating conditions (FC = 7.13 average value), high level of intention to use (ITU = 10.13 average value), high level of perceived adaptability (PAD = 11.00 average value), high level of perceived enjoyment (PENJ = 20.37 average value), the highest level of perceived ease of use (PEOU = 19.00 average value), high levels of perceived sociability (PS = 14.37 average value), perceived usefulness (PU = 11.25 average value), social influence (SI = 8.25 average value), and social presence (SP = 12.25 average value). The confidence level was low (TRUST = 6.37 average value) due to the short time of interaction of the participants with the robot and the low number of participants in the study.

However, the participants reported a high level of usability of the robot solution, with an average value of the SUS score greater than 70.

The five key elements of human-robot interaction were analyzed using the Godspeed questionnaire. The result was a good level of anthropomorphism (ANTRO = 3.13 average value), animacy (ANIM = 3.37 average value), likeability (SIMP = 4.50 average value), perceived intelligence (INT = 4.00 average value), and perceived safety (SICUR = 3.87 average value).

The participants confirmed a good level of agreement in the UEQ domains: Attractiveness (1.60 average value), Efficiency (1.15 average value), Perspicuity (1.50 average value), Dependability (1.00 average value), Stimulation (1.13 average value), and Novelty (1.78 average value).

In the second part of the study, as shown in Table 6, the patients reported a moderate level of perceived stress when they could use the robot for remote visits. This is based on an average PSS score of 18.17. 

In the same context, the participants reported a high level of usability of the robot solution (SUS > 70 average value) and confirmed a good level of agreement in the UEQ domains: Attractiveness (1.55 average value), Efficiency (1.63 average value), Perspicuity (1.42 average value), Dependability (1.29 average value), Stimulation (1.33 average value), and Novelty (1.67 average value). 

As shown in Table 7, after the interaction with the robot in the second part of the study, the level of perceived stress of the healthcare operators is low (PSS mean score = 11.50). The healthcare operators reported a good level of agreement in the UEQ domains: Attractiveness (1.37 average value), Efficiency (1.19 average value), Perspicuity (1.37 average value), Dependability (1.37 average value), Stimulation (1.31 average value), and Novelty (1.37 average value).

Data on family members are not available because the patients decided not to fill out the questionnaires.

## 4. Discussion

The piloting of the robotic solution was conducted in an intensive care unit. The primary objective of the study was to reduce the circulation of pathogens; in addition, we wanted to evaluate the acceptability of the robot among the users. We tried to make the robot as friendly and acceptable as possible for the staff and the patients. The results of the test showed a high level of acceptability in the AMQ domains, usability of the robot solution in the SUS questionnaire, positive overall perceptions of the robot in the Godspeed questionnaire, and a good level of agreement in the UEQ domains.

We adopted a state-of-the-art approach to analyze the acceptability of robotic technologies for humans and to explore the points of contact between the areas of human-robot interaction (HRI) and human-centred design (HCD). A “human-centred HRI” approach seems to be the most appropriate: it is based on the analysis of the characteristics (physical, morphological, behavioral, etc.) that the robot must have in order to be acceptable, efficient, and pleasant for humans. The challenges in this area mainly concern the search for a balanced and coherent design between behavior and appearance of the robot, the designing of socially acceptable behaviors, and the development of new methods and tools for designing and evaluating HRI, identifying the needs of individuals and groups of subjects to whom a robot could adapt and respond, avoiding the uncanny valley [10], etc. HRI methods focus mainly on two elements: the first concerns actual acceptability, i.e., all those factors that influence the intention to use a robot (e.g., ease of use, enjoyment, controllability, etc.), evaluated extensively through various models of acceptance of the technology (for example, Almere); the second concerns usability, defined as the effectiveness, efficiency, and satisfaction with which users achieve specific goals in specific environments [10] (ISO 9241-11). Within HRI, this has been translated into research on how various specific factors may influence the acceptance of the robot by the users, such as morphological aspects [11], facial and affective expressions [12], linguistic and cultural differences [13], behaviors [14], personal space [15], or other variables such as age or education [16].

From the analysis of the studies present in the literature, it is evident that there is no distinction between general opinions about robots and user perceptions during the actual interaction with the robots. Most of the studies in the field of HRI investigate elements such as cultural differences, attitudes, and feelings towards robots. Beliefs and personal opinions can influence acceptance by the users, affecting the quality of human-robot interaction. These aspects can be measured starting with a series of qualitative and quantitative data collected on different groups of people. Designing acceptability for HRI means applying guidelines and principles in the early design phases of the robot; this implies considering both the data relating to the user (user-centered) and all the other elements (context of use, anxiety, fear or attitudes towards robots, social opinions, or beliefs, ethical or legal aspects, etc.) that can influence the success of human-robot interaction.

Specifically, regarding social and assistive robots, studies that involve users during the design process have shown excellent results in terms of overall user experience and acceptance of the robotic technology [17,18,19].

The robot has also been used to make teleconferences with families and clinical professionals. In addition, in this case, the results show a moderate reduction in perceived stress, a high level of usability of the robotic solution in the SUS questionnaire, and a good level of agreement in the UEQ domains.

In line with the analysis of the main assistive robots (commercial and non-commercial) tested in the literature, it can be deduced that the main activities for which they are designed concern three fundamental aspects of assistance: functional (linked to help during daily activities in domestic environments); social (aimed at counteracting the danger of isolation and depression of the patient), such as telepresence, entertainment, communication with other people or company to relieve stress, promote the expression of emotions or internal states and increase both the individual’s sense of autonomy and security; therapeutic (linked to the administration of therapies both for problems or physical and psycho-cognitive pathologies) to relieve stress and manage emotions, support, and stimulate physical activity.

The activities performed by robots influence their relationship with patients, determining a greater or lower acceptance or a greater or lower willingness to prolong use over time. We can find a match between the different types of activities identified and described above and the type of acceptance. The latter can be functional [17,20,21] or social/therapeutic [22,23].

The first is understood in terms of utility (PU), ease of use (PEOU), and usability, which represent those pragmatic qualities determining the attitude (ATT) of people towards robots [24,25]. Functional acceptance is also closely linked to aesthetic aspects. In fact, the robot’s morphology should be as faithful as possible to the actions that it is able to perform and the skills it possesses; in fact, a mismatch can negatively affect the PEOU and the pleasure of use (PENJ). Furthermore, the effectiveness and efficiency with which the robot completes specific activities determines its credibility and trust (Dependability) towards the system [26,27].

The effectiveness of an assistive robot that carries out social and therapeutic activities depends a lot on the perception of how intelligent (perceived intelligence) or secure (perceived security) the system is, as well as how much it is able to adapt (PAD) to the needs of the people who use it [17,28]. Furthermore, the emotional and fiduciary relationship that is established between a robot and a human being, especially in the case of pet robots, for social communication or affective therapy depends on contextual factors such as social norms (SI), anxiety about robots (ANX), or past experiences (related experiences), as well as hedonic factors such as attractiveness (Attractiveness) or the level to which users believe that the robot behaves realistically (Realism) [29,30,31,32].

Based on these considerations and on the data collected during the research, we can confirm the choice we made of a robot that can fit well for patients admitted to a department such as the Hematological Intensive Care Unit, where hospitalized patients are characterized by a high level of frailty and must be isolated for the entire duration of their stay.

Clearly, there are limitations to this study. In the first phase of the study, a psycho-behavioral evaluation after the interaction with the robot was made in order to avoid polarization of the results due to the stress linked to the completion of all the questionnaires. Since we were more interested in the evaluation of the perception of the robot, we preferred to make the psycho-behavioral evaluation at baseline. In the second phase of the study, a baseline assessment of perceived stress was not made because of the short time elapsed between the choice of the patient and the patient visit. The small number of patients involved in the study limited the statistical power of the results. This is mainly due to the short duration of the study, the long length of stay of the patients within the Hematological Intensive Care Unit, and the low compliance with the inclusion criteria.

## 5. Conclusions

The aim of the SYRIACA project was to test the capability of a robot to take care of repetitive tasks, thus reducing the exposure of patients and caregivers to risks related to healthcare-associated infections. The robot was tested in four different pilot sites. In this paper, the results of the experimentation about the acceptability of the robot in an Intensive Hematological Unit are presented and examined. The results of the experimentation at the pilot site, Fondazione IRCCS Casa Sollievo della Sofferenza, indicate a high level of acceptability of the robot by both patients and healthcare operators. The various functions of the robot, including logistics, surveillance, entertainment, and remote visits, were well-regarded and aroused curiosity among users. Patients expressed interest in interacting with the robot multiple times, highlighting its potential for engagement and the delivery of beneficial experiences. The success of the robot in the pilot study can be attributed to the human-centered approach in designing the human-robot interaction. By considering user needs, behaviors, and context of use, the robot was developed to be socially acceptable and efficient.

The robot’s involvement in remote visits with families and clinical operators was found to contribute to a moderate reduction in perceived stress among the patients. The robot’s presence and interactive capabilities served as a means to alleviate feelings of isolation and enhance communication, promoting overall well-being among patients. The successful implementation of the robot in a Hematological Intensive Care Unit had significant implications for healthcare settings during the COVID-19 pandemic and beyond. By engaging in repetitive and potentially risky tasks, the robot can assist patients and healthcare professionals and reduce their exposure to pathogens, ensuring a safer working environment. The study acknowledges some limitations, such as the lack of psycho-behavioral evaluation after the interaction with the robot in the first phase and the absence of a baseline assessment of perceived stress in the second phase, as well as a limited statistical power due to the low number of recruited patients. Future research could address these limitations and further explore the long-term impact of the robot on patients’ well-being and healthcare professionals’ work experiences.

## Figures and Tables

**Table 1:** Patient age distribution.

Phases	Patient ID	Age
1	23-1	19
	23-2	55
	23-3	56
	23-4	28
	23-5	31
	23-6	34
	23-7	64
	23-8	66
2	23-9	64
	23-10	66
	23-11	50
	23-12	61

**Table 2:** Patient smartwatch usage duration.

Patient ID	Smartwatch Usage Duration
23-1	3 weeks
23-2	1 week
23-3	2 weeks
23-4	2 weeks
23-5	3 weeks
23-6	3 weeks
23-7	1 week
23-8	1 week

**Table 3:** Patient remote visits.

Patient ID	Family	Dietician	Psychologist
23-9		X	X
23-10		X	
23-11	X		X
23-12	X		

**Table 4:** Patients’ baseline evaluation.

Test	Patients (*n* = 8)
NPI	1.75 ± 1.28
HDRS	2.00 ± 2.39
EQ-5D-3L	0.63 ± 0.92
EQ VAS	68.50 ± 15.66

**Table 5:** Test results after patient interaction with the robot.

Test	Domains	Patients (*n* = 8)
**AMQ**	ANX	18.13 ± 2.94
	ATT	12.25 ± 1.48
	FC	7.13 ± 1.55
	ITU	10.13 ± 3.18
	PAD	11.00 ± 2.33
	PENJ	20.37 ± 2.87
	PEOU	19.00 ± 2.56
	PS	14.87 ± 3.44
	PU	11.25 ± 1.90
	SI	8.25 ± 0.88
	SP	12.25 ± 3.61
	TRUST	6.37 ± 1.99
**SUS**	-	70.63 ± 8.73
**GOODSPEED**	ANTRO	3.13 ± 0.64
	ANIM	3.37 ± 0.74
	SIMP	4.50 ± 0.75
	INT	4.00 ± 0.75
	SICUR	3.87 ± 0.64
**UEQ**	Attractiveness	1.60 ± 0.94
	Efficiency	1.15 ± 1.02
	Perspicuity	1.50 ± 0.93
	Dependability	1.00 ± 0.57
	Stimulation	1.13 ± 0.95
	Novelty	1.78 ± 0.83

**Table 6:** Patient evaluation in the second part of the study.

Test	Domains	Patients (*n* = 4)
**PSS**	-	18.17 ± 7.81
**SUS**	-	87.85 ± 23.16
**UEQ**	Attractiveness	1.55 ± 1.06
	Efficiency	1.63 ± 0.92
	Perspicuity	1.42 ± 0.97
	Dependability	1.29 ± 1.09
	Stimulation	1.33 ± 1.23
	Novelty	1.67 ± 0.86

**Table 7:** Healthcare operator evaluation in the second part of the study.

Test	Domains	Healthcare Operators (*n* = 4)
**PSS**	-	11.50 ± 4.79
**UEQ**	Attractiveness	1.37 ± 1.59
	Efficiency	1.19 ± 1.39
	Perspicuity	1.37 ± 1.63
	Dependability	1.37 ± 1.45
	Stimulation	1.31 ± 1.23
	Novelty	1.37 ± 1.45

## Data Availability

Paper-based questionnaire answers are not available due to privacy reasons.
